# Sympatric Speciation: When Is It Possible in Bacteria?

**DOI:** 10.1371/journal.pone.0053539

**Published:** 2013-01-17

**Authors:** Jonathan Friedman, Eric J. Alm, B. Jesse Shapiro

**Affiliations:** 1 Program in Computational and Systems Biology, Massachusetts Institute of Technology, Cambridge, Massachusetts, United States of America; 2 Department of Civil and Environmental Engineering, Massachusetts Institute of Technology, Cambridge, Massachusetts, United States of America; 3 Department of Biological Engineering, Massachusetts Institute of Technology, Cambridge, Massachusetts, United States of America; 4 Broad Institute, Cambridge, Massachusetts, United States of America; 5 Center for Communicable Disease Dynamics, Harvard School of Public Health, Boston, Massachusetts, United States of America; 6 Department of Organismic and Evolutionary Biology, Harvard University, Cambridge, Massachusetts, United States of America; Uppsala University, Sweden

## Abstract

According to theory, sympatric speciation in sexual eukaryotes is favored when relatively few loci in the genome are sufficient for reproductive isolation and adaptation to different niches. Here we show a similar result for clonally reproducing bacteria, but which comes about for different reasons. In simulated microbial populations, there is an evolutionary tradeoff between early and late stages of niche adaptation, which is resolved when relatively few loci are required for adaptation. At early stages, recombination accelerates adaptation to new niches (ecological speciation) by combining multiple adaptive alleles into a single genome. Later on, without assortative mating or other barriers to gene flow, recombination generates unfit intermediate genotypes and homogenizes incipient species. The solution to this tradeoff may be simply to reduce the number of loci required for speciation, or to reduce recombination between species over time. Both solutions appear to be relevant in natural microbial populations, allowing them to diverge into ecological species under similar constraints as sexual eukaryotes, despite differences in their life histories.

## Introduction

Microbes have adapted to nearly every ecological niche imaginable on earth, yet the evolutionary mechanisms of the specialization process and their constraints remain poorly understood. In our recent study of ecological differentiation between two marine *Vibrio* populations [1], we were surprised to observe relatively few regions of the genome underlying differentiation: up to 11 (but as few as 3) in the ‘ore’ genome, with different alleles fixed between large (L) and small (S) particle associated strains, and up to 19 in the ‘flexible’ genome (horizontally transferred tracts of DNA exclusively present in genomes from one habitat but absent in the other). Habitat-specific alleles showed extraordinarily high sequence divergence compared to other parts of the genome, yet had very low levels of within-habitat polymorphism [1]. Thus, it is likely that these alleles arrived recently by recombination with other more distantly related populations and spread rapidly within S or L populations before many polymorphisms could arise by mutation, suggesting that recombination rather than mutation is the dominant source of genetic variation. Aside from these few habitat-specific regions, most of the genome showed a history of rampant recombination within and between populations (as evidenced by different genealogies for roughly every gene in the genome), consistent with a relatively large influence of recombination on *Vibrio* genomes [2]. At face value, this observation seemed to be satisfactorily explained by modeling work predicting that speciation by habitat shift should not involve many loci [3]–[7]. However, these were models of sexual eukaryotes, which – unlike bacteria – necessarily undergo homologous recombination every generation and form species by assortative mating and sexual isolation. In their 1986 paper, “Sympatric speciation: when is it possible?”, Kondrashov and Mina [8] conclude that for sexual populations “sympatric speciation is possible when essential differences (including isolating mechanisms) between the formed species depend on up to 10 loci”. Here, we reformulate these classic models in order to ask the same question for bacteria: when, and with how many loci involved, is sympatric ecological differentiation likely to occur?

While many different speciation scenarios are possible for bacteria, here we limit ourselves to one scenario that we hypothesize to be most relevant for *Vibrio* and other frequently recombining bacteria, where populations differentiate in sympatry, with relatively high rates of recombination among all individuals. For simplicity, we take sympatry to mean that all individuals recombine with equal probability, regardless of their niche or species affiliation. This is, intentionally, a rather extreme version of sympatry, in which there is no opportunity for establishment of new species in physical isolation. Although extreme, such a model is consistent with the suspected transience of marine *Vibrio* microhabitats: vibrios disperse rapidly among invertebrate hosts [50], and particle habitats may have short half-lives. Rapid turnover of microhabitats would allow very little opportunity for recombination within habitats (“environmental assortative mating,” as termed by J. Mallet, personal communication). If turnover is less rapid, a model of ‘mosaic sympatry’ [9] may be more appropriate for bacteria with preferences for different microhabitats (*e.g.* large zooplankton or copepods vs. small organic particles), but with frequent mixing among microhabitats. More frequent temporal sampling of marine vibrios will be necessary to decide which type of model is most realistic. For now, we limit ourselves to exploring the fully sympatric model; in the future, the model could be expanded to incorporate mosaic sympatry. We do not aim to describe bacteria that speciate due to strong barriers to recombination (allopatry, *e.g.* [10]), or in which recombination is weak relative to mutation, selection and drift [11]–[13]; such ‘effectively clonal’ populations are already well described by various versions of the stable ecotype model, involving periodic selection and genomewide selective sweeps [11], [14].

Sympatric speciation is increasingly being observed in plants and animals [7], [15]. In all domains of life, the likelihood of sympatric speciation depends on the balance between the homogenizing force of recombination (inhibiting speciation) and disruptive selection, favoring speciation by adaptation to different niches [16]. This balance – essentially a restatement of Haldane’s isolation theory [17] – can be applied to individual genes, such that some parts of the genome (*i.e.* those under disruptive selection) can be strongly differentiated, while others (neutral loci) are not. Here we are interested in the early stages of speciation, in which populations become differentiated at selected loci, but not necessarily at neutral loci [18]. This amounts to an ‘ecological’ species concept, in which species are defined as genotypes optimally adapted to different niches through acquisition of selectively favored alleles at one or more loci [19]. Speciation is therefore driven by selection on adaptive loci, and does not require differentiation in neutral loci, or any form of assortative mating. For the purposes of this study, we define speciation in this ecological sense: directional selection favors different alleles in different niches. As a result, incipient species are defined as having the optimal combination of alleles in their respective niches. For modeling purposes, a stable optimum can be defined because the fitness landscape is controlled; this is, of course, a simplification of natural habitats, in which many local maxima may exist, and the fitness landscape may be constantly changing.

Following Kondrashov [3], [4], [8], sympatric speciation can be conceptually divided into early stages, where recombination helps generate optimally adapted genotypes, and late stages, where recombination homogenizes incipient species unless barriers to gene flow emerge. At early stages, the rate of adaptation to new niches may be increased dramatically in recombining (as opposed to strictly clonal) populations by bringing multiple adaptive alleles into a common genomic background [20], and unlinking them from deleterious mutations [21]. When fitness in a new niche is controlled by adaptive mutations that can fix in any order, recombining populations are favored by selection [22], but when fitness depends on the order of fixation, recombining populations adapt more slowly than clonal populations [23]. Sex may also come with a cost (*e.g.* reduced rate of cell division), in which case switching between sexual and clonal phenotypes provides an optimal strategy [24].

Although recombination speeds up adaptation within a single population (at least when fitness is additive), it can be a powerful hindrance to the later stages of sympatric speciation. In the absence of barriers to gene flow between emerging species, recombination may homogenize their gene pools, effectively preventing speciation. Under the biological species concept [25], assortative mating is generally required for sympatric speciation, and this is borne out in modeling work [5], [26]. In these and earlier models [4], [8], sympatric speciation was delayed by increasing the number of loci contributing to assortative mating, such that speciation is very unlikely when more than ∼10 loci are involved.

In support of this theoretical prediction, recent surveys of genomic variation in sympatric pairs of sexual eukaryotic species have identified just a few (<10) genomic ‘islands’ of high divergence between mosquito species [27]–[30]. These islands could contain genes responsible for ecological specialization, assortative mating, or both. Sequence divergence in islands may locally inhibit recombination between incipient species, extending the islands into ‘continents’ of divergence in a process termed ‘divergence hitchhiking’ [31]. Similarly, in stickleback fish, a small number of alleles (∼1–5) involved in pelvic morphology [32] and armor plate patterning [33] are sufficient for significant ecological differentiation. Although these alleles may have contributed to early divergence of marine and freshwater stickleback species ∼2 million year ago, many more adaptive changes (in ∼90–215 loci) have occurred since that time [34].

With or without assortative mating, it seems plausible that speciation will proceed more readily when fewer loci are required to confer ecologically different phenotypes to each nascent species. This concept is explained eloquently by Kondrashov [4]: *“In a panmictic equilibrium population the phenotype variance* [and the variance in relative fitness, if this phenotype is related to fitness] *decreases with the growth of the number of loci controlling any character. It leads to the weakening of the effects of selection […].”* Under an additive fitness model where each locus contributes equally to niche adaptation, as more loci are involved in adaptation, the fitness landscape becomes ‘smoother’ and the competitive advantage of the ‘optimal’ genotypes becomes relatively weaker, such that intermediate genotypes are maintained and speciation is impeded. However, this phenomenon has not yet been thoroughly explored in a microbial context, with asexual reproduction and variable recombination rates.

Regardless of what type of fitness model is used, as more loci are necessary to achieve an optimal genotype (ecological species), more recombination will be needed to collect the optimal allelic combination into a single genome. However, high recombination rates come at a cost: once an optimal genotype is generated for a new niche, there is nothing to prevent it from recombining with individuals from the ancestral niche, thus delaying or preventing speciation. We investigate the importance of this apparent tradeoff in a microbial context, where recombination can occur, but not necessarily every generation. We strove for the simplest possible model that could account for our empirical findings that few adaptive loci could drive ecological differentiation in highly recombining bacterial populations [1]. Like Kondrashov’s models, ours is a deliberate abstraction aimed at capturing major qualitative features of speciation. We follow the conceptual framework of Kondrashov [8], changing details as necessary to suit bacterial populations. Unlike models of sexual eukaryotes, we use a model without assortative mating, which we assume to be negligible among very closely-related bacteria in the very early stages of speciation. Although forms of assortative mating cannot be ruled out, studies of highly recombining bacteria such as *Streptococcus pneumoniae* have failed to find evidence for assortativeness [35]. However, because recombination drops loglinearly with sequence divergence [12], [36]–[39], barriers to gene flow should eventually emerge between more distantly-related bacteria, and even very closely related sympatric bacterial populations show signs of emerging barriers to gene flow [1], [40]. We chose not to model assortativeness or any barriers to gene flow for two reasons. First, such models have already been explored [5], [8], [26]. Second, we are interested in the early stages of speciation, which are necessary for, yet do not guarantee the establishment of, long-lived species. These early stages are driven by directional selection, and may or may not be followed by reduced gene flow between incipient species.

## Model

Our sympatric speciation simulation (*symsim*, implemented in MatLab and available at http://almlab.mit.edu/symsim.html) models a microbial population growing in an environment composed of two distinct niches, one ancestral (niche 0) and one derived (niche 1). Niches are deliberately abstract, but could consist of different carbon sources, optimal growth temperatures, host or particle associations, etc. Genotypes consist of *L* unlinked adaptive loci, each with two allelic states, 0 or 1, conferring adaptation to niche 0 or 1, respectively, and one representative ‘background’ locus with two allelic states both neutral to fitness.

**Figure 1:**
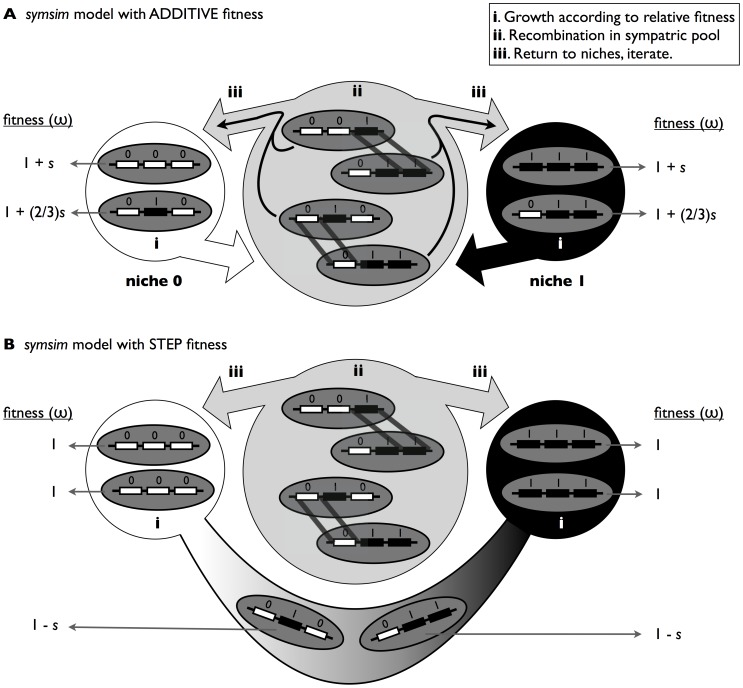
Schematic of the *symsim* model. (A) Additive fitness. The steps of the simulation are (i) growth/selection according to relative additive fitness within each niche, (ii) small probability of recombination (*r*) by gene conversion of homologous loci (diagonal lines) in a sympatric, mixed pool of genotypes from both niches, and (iii) individuals return to the niche to which their genotype is best adapted (*e.g.* in this 3-locus example, genotypes 000 and 010 go to niche 0, while 111 and 011 go to niche 1). Steps (i), (ii) and (iii) are iterated for a set number of generations or until any of the derived alleles go extinct. (B) Step fitness. The steps of the simulations are the same as for additive fitness, except that individuals can grow and be selected in both niches. Optimally adapted genotypes (111 and 000) compete in just one niche. Intermediates compete in both niches, but pay a fitness cost *s* for niche switching. In the example shown, an individual of genotype 010 obtains 2/3 of its resources in niche 0 (and adds a count of 2/3 of an individual to the population size of niche 0), and 1/3 from niche 1 (and adds a count of 1/3 of an individual to niche 1).

We considered two models of fitness: one additive and the second a ‘step’ function [8]. In both models, fitness is a function of *f_i,j_*, the fraction of adaptive loci in genotype *i* that contain alleles adapted to niche *j*. Optimally adapted genotypes (defined here as ecological species) have *f_i,j_* = 1. In the additive model, microbes compete and reproduce exclusively in the niche to which their genotype is best adapted (Figure 1A), and fitness is an additive function of *f_i,j_*. For example, with *L* = 5, the genotype 11100 would compete in niche 1 with *f_11100, 1_* = 3/5. Genotypes always reproduce (by clonal cell division) in the niche for which *f_i,j_* >0.5; if *f_i,j_* = 0.5, a niche is chosen at random. This constitutes a strong tradeoff in niche adaptation: each strain can only have one niche. As a result, until a strain acquires at least *L*/2 niche-1 adaptive alleles, it will compete in niche 0, with any niche-1 alleles incurring a fitness disadvantage. The step fitness model relaxes this strong tradeoff: strains with *f_i,j_* <1 may shuttle between niches, acquiring their full complement of resources from a combination of both niches (Figure 1B). However, the disruptive selection necessary for speciation is maintained because all intermediate genotypes must pay a fitness cost for the time and energy spent switching between niches. This cost (in the form of a selective coefficient, *s*) is uniform for all *f_i,j_* <1. Thus, all intermediates pay an identical fitness cost relative to the specialized, optimally niche-adapted genotypes.

Both fitness models, while simple, are reasonable starting points for describing natural bacterial populations with different metabolic or microhabitat specializations. In natural marine *Vibrio* populations, for example, ecological differentiation is likely driven by genes involved in host or particle attachment (*mshA* and *syp* genes) and transcriptional regulators (*sypG*, *rpoS*) [1]. Additive fitness effects would result if there were an incremental advantage to encoding and expressing each additional adaptive gene or allele. Alternatively, intermediate genotypes could gather resources from two different hosts or particle types, but with a penalty for time spent switching between the two, as in the step model. Similar fitness landscapes can be imagined for populations that undergo ecological differentiation based on different metabolic or successional strategies [41].

*Symsim* is a discrete generations model with varying population size, required for investigating the colonization of a new, initially empty niche. Each generation consists of resource competition, population growth and recombination, as follows:

### I. Competition

At each generation, each niche has an amount of resource *R* for which individuals compete.

In each niche, the resource is partitioned among individuals according to their competitive fitness. In the additive model:
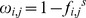
(1a)


And in the step model:
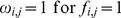
(1b)

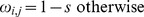
where *s* is a tunable parameter controlling the strength of selection. We ran simulations using *s* = 0.1 and 0.01, representing the range of selective coefficients measured for single fixed beneficial mutations in experimentally evolved *E. coli* populations [42]. Instead of selection on a single locus, in our model *s* stands for disruptive selection (Equation 1a) or negative selection (Equation 1b) on multiple loci. The average amount of resource allocated to each individual of genotype *i* in niche *j* scales with their relative fitness:
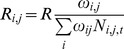
(2)where *N_i,j,t_* is the number of cells with genotype i in niche j at time t. Under additive fitness, genotypes only obtain resources in the niche to which they are best adapted (for which *f_i,j_* ≥0.5, as described above). Under step fitness, each individual can obtain resources from both niches, and also counts toward the number of cells in both niches, proportionally to its genotype. For example, genotypes with *f_i,j_* = 0 obtains resources only from niche 0, *f_i,j_* = 1 only from niche 1, and *f_i,j_* = 0.5 obtains half its resources from each niche and contributes half a count to each population size.

### II. Clonal Reproduction

At each generation, the average expected number of offspring of each individual of genotype *i* in niche *j* is:
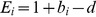
(3)where *d* is the death rate and *b_i_*, the birth rate, is a saturating Michaelis-Menton function of the resources allocated to it:
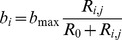
(4)where *b_max_* is the maximal birth rate per cell and *R_0_* is the half-saturation constant of the birth function. Therefore, the number of individuals (*N*) of genotype i in the next generation is drawn from a Poisson distribution:



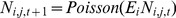
(5)The carrying capacity (*K*) of each niche (the steady-state population size) is related to the amount of resource available and the birth and death parameters by:
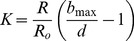
(6)


In all simulations, we set *R* = 1, *b_max_* = 10, *d* = 0.1, and *K* = 10^6^ in each niche. We investigated two different selective coefficients: *s* = 0.1 and *s* = 0.01.

### III. Recombination

After population growth and selection, all microbes enter a common sympatric pool and recombine with one another at random, independently of their genotype (Figure 1), with rate *r* per individual per locus. The total number of recombination events per generation is therefore *r* × *N* × (*B*+*L*), where *N* is the total number of individuals in both niches, *L* is the number of adaptive loci, and *B* is the number of neutral background loci (set at *B* = 1 in all simulations). In each event, a donor/acceptor pair of microbes is chosen at random, and one of the *B*+*L* genomic loci is chosen to be recombined, also at random. Recombination between homologous loci is non-reciprocal, and occurs by gene conversion resulting in allelic replacement of the acceptor by the donor allele.

Cycles of growth and recombination continue for a set number of generations, or until any of the adaptive alleles go extinct (meaning that speciation fails to occur). Mutation is not included in our model because we are particularly interested in the case where recombination rates are much higher than mutation rates, and where adaptive alleles are highly divergent, containing multiple nucleotide differences. We also point out that *symsim* is a model of homologous recombination by gene conversion, although extending it to include non-homologous recombination (horizontal gene transfer) would be straightforward.

## Results

We aimed to answer two questions: (1) How long does it take the derived (niche-1 optimized) species to appear, and what is the likelihood that it appears at all? (2) Given that the derived species does appear, what is its equilibrium frequency relative to intermediate (sub-optimal) genotypes? In other words, what is the ‘completeness’ of the ecological speciation process?

### 1. Recombination Expedites the Appearance of New Ecological Species

**Figure 2:**
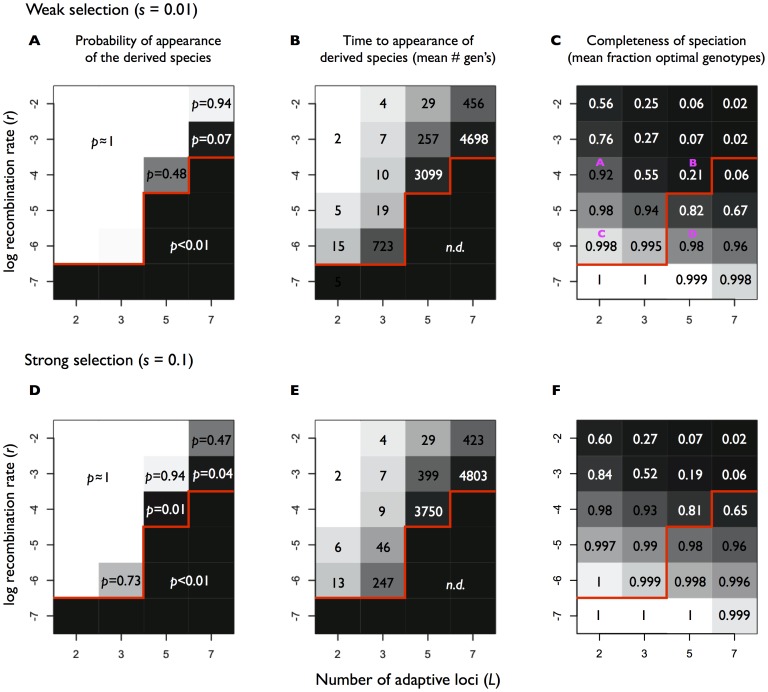
Results of *symsim* model under additive fitness. (A, B, C) Weak selection. (D, E, F) Strong selection. (A, D) Probability of appearance (*p*) of the derived (niche 1) optimal genotype in 100 replicate simulations for each combination of the number of loci involved in niche adaptation, *L* and the recombination rate, *r*. High probabilities (*p* = 1) are shown in white, low probabilities in black, and intermediate probabilities in grey scale. The space under the red line indicates extinction of the niche-1 optimal genotype in all 100 replicates (*p*<0.01). (B, E) Time to appearance of the niche 1 optimal genotype (mean over 100 replicate simulations). The red line is the same as in (A); *n.d.* refers to appearance time not determined, or effectively infinite, because extinction of niche-1 alleles occurred before the optimal genotype could appear. Shorter times are shown in white, effectively infinite times in black, and intermediate times in grey scale. (C, F) Completeness of speciation. The mean fraction of the pooled populations (niche 0 and 1) occupied by optimally-adapted genotypes is based on 10 replicate simulations for every combination of *L* and *r*. Complete speciation (optimal genotype fraction near 1) shown in white, incomplete in black, and intermediate completeness in grey scale. Magenta letters in C refer to the same simulations depicted in panels (A, B, C, D) of Figure 4.

**Figure 3:**
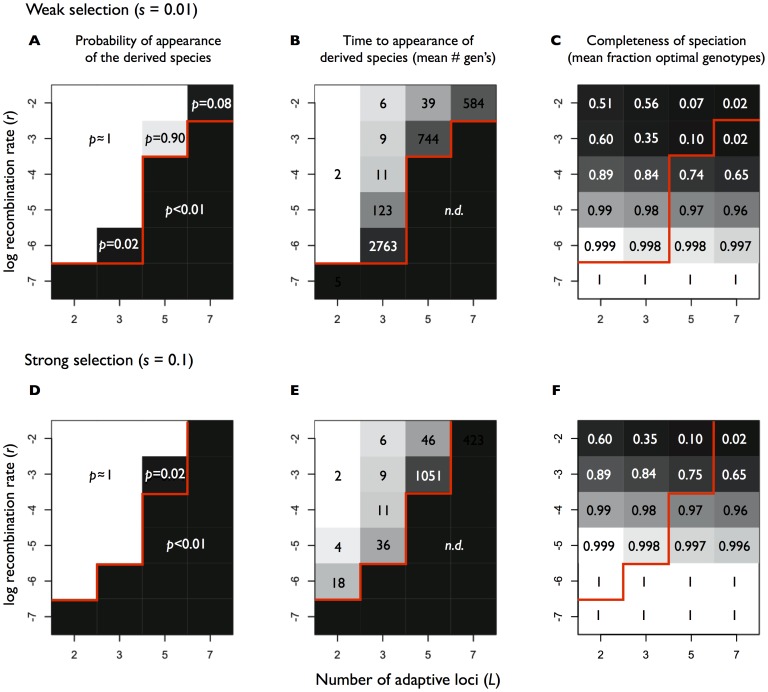
Results of *symsim* model under step fitness. (A, B, C) Weak selection. (D, E, F) Strong selection. See Figure 2 legend.

To address the first question, we initiated the *symsim* model with niche-1 empty (the novel niche having just appeared) and niche-0 (the ancestral niche) occupied by 95% optimal genotypes. The remaining 5% of the population contained a niche-1 allele at a single locus (distributed uniformly across the *L* loci), with all other loci containing niche-0 alleles. We varied the model of selection (additive or step), the selection coefficient (*s*), the number of loci involved in niche adaptation (*L*) and the recombination rate (*r*), and allowed the simulation to proceed until the niche-1 optimal genotype (defined as the derived species) was generated by recombination, or until any of the niche-1-adapted alleles went extinct, rendering the niche-1 optimal genotype unattainable. We performed 100 replicate simulations for each parameter combination and obtained qualitatively similar results for both additive (Figure 2) and step (Figure 3) models of selection, with both strong and weak selection. We will therefore focus first on the results of the additive model with weak selection (*s* = 0.01) because they are also representative of the qualitative features of the other models.

How does the number of loci contributing to adaptation affect the likelihood that the derived species is generated by recombination? With only two adaptive loci, the derived optimal genotype is always generated by recombination (Figure 2A), even when the recombination rate is relatively low (*r* = 10^−6^, resulting in only ∼2 recombination events per locus per generation in the pooled population), but not when it approaches zero expected recombination events per generation (*r* = 10^−7^). As the number of adaptive loci is increased to *L* = 3, the derived species still almost always appears, but takes longer to do so. With *L* = 5, a higher recombination rate (*r* = 10^−4^) is required for even a 48% chance of appearance before extinction, and with *L* = 7 appearance is only likely at very high recombination rates (*r* = 10^−2^).

The exact values of *L* and *r* required for appearance of niche-1 optimal genotypes depend on the selection model, strength of selection and population size (which influences the likelihood of stochastic extinctions, not investigated here). With more than 2 adaptive loci, the appearance of derived optimal genotypes is less likely under the step fitness model (Figure 3), likely due to strong and uniform selection against intermediate genotypes. To a lesser extent, increasing the strength of selection also hinders slightly the appearance of derived optimal genotypes (compare panels A and D in Figures 2 and 3); the effect is only slight because fitness is relative to other competitors in the population (Equation 2). In general, the simulations all support a major qualitative conclusion: higher recombination rates are necessary for ecological speciation when more adaptive loci are involved. For many adaptive loci and low recombination rates (area below the red line in Figures 2 and 3), ecological speciation is very unlikely to be initiated.

### 2. Recombination Hinders the Later Stages of Speciation

To investigate any potential tradeoffs between the early and late stages of speciation (appearance of the new species, and disappearance of sub-optimal intermediate genotypes, respectively), we ‘fast-forwarded’ the simulation to a point in time when the derived species (niche-1 optimal genotype) had reached 1% of the pooled population (having thus escaped extinction by drift), the ancestral (niche-0) species constituted 95%, and the intermediate genotypes were distributed uniformly to make up the remaining 4%. From these starting conditions, we ran *symsim* for 100,000 generations for each combination of *L* and *r*. In each replicate simulation, we recorded the maximum mean frequency of optimal genotypes (averaged over both niches) observed any time after 25,000 generations. Based on visual inspection, genotype frequencies always reached an equilibrium by this point, so the maximum frequency of optimal genotypes proved to be a reasonable measure of the ‘completeness’ of speciation.

We found that the high recombination rates necessary to generate the derived species tended to hinder the completion of speciation later on. For example, under additive fitness and *s = *0.01, with *L* = 5 adaptive loci, a minimum of *r* ≈10^−4^ is required for the derived species to appear and survive (Figure 2A,B), yet this amount of recombination also generates many intermediate genotypes, resulting in a low equilibrium frequency (0.21) of optimally adapted species (Figure 2C, 4B). The incompleteness of speciation at high recombination rates is less pronounced with fewer adaptive loci, for example when *L* = 2 (Figure 2C, 4A). When the recombination rate is kept low (*r* = 10^−6^), speciation proceeds essentially to completion, with nearly the entire populations of both niches occupied by optimally adapted genotypes (Figure 2C, 4C,D). Of course, such low recombination rates would be unlikely to produce optimally adapted genotypes in the first place (falling below the red line in Figures 2), resulting in a serious impediment to sympatric speciation as increasing numbers of adaptive loci are involved.

**Figure 4:**
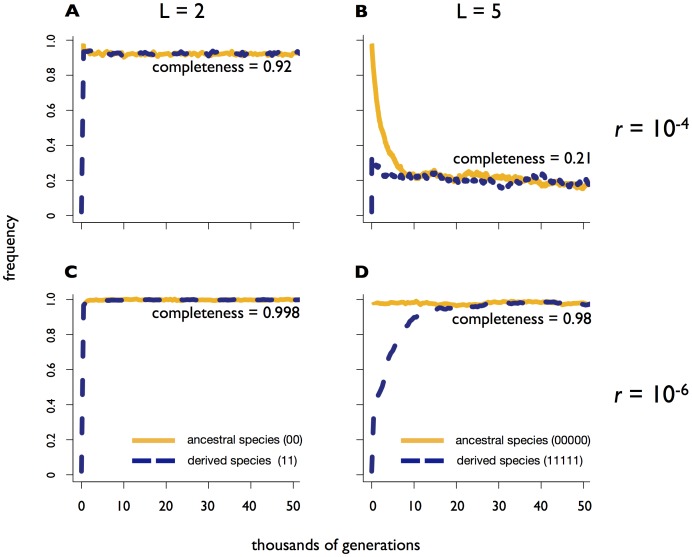
Higher recombination rates maintain intermediate genotypes and reduce the completeness of speciation. Panels A, B, C and D show dynamics of a single simulation under different combinations of *L* (number of adaptive loci) and *r* (recombination rate), corresponding to magenta letters in Figure 2C. The y-axis shows the frequency of optimal genotypes in a given niche (ancestral or derived).

The same tradeoff between early and late stages of speciation is also observed in the step fitness model. Although speciation is generally more complete under step than additive fitness (for the same values of *r* and *L*), the combinations of *r* and *L* most likely to maintain complete speciation are unlikely to have generated optimally adapted derived genotypes at earlier stages (Figure 3). Thus, even under a step fitness model, in which the variance in fitness does not decrease with *L* (and there is no weakening of selection with *L*, as in the additive fitness model), we still observe a tradeoff between early and late stages of speciation.

### 3. A Practical Note on our Ability to Recognize Adaptive Loci

Sympatric species of bacteria are difficult to recognize in the wild because the niches to which they adapt are often difficult to observe, and their adaptive genomic changes potentially indistinguishable from neutral changes. In practice, sympatric ecological species can be identified when different alleles have been fixed between populations by directional selection [43], [44]. In a perfectly clonal scenario (*r* = 0), selectively neutral ‘background’ alleles would also become differentially fixed between species, making them hard to distinguish from the adaptive alleles driving speciation.

Our simulations included a representative neutral background locus, and we asked under what circumstances it could be mistaken for an adaptive locus. Selection acts to fix a different allele in each niche at adaptive loci, but not background loci. However, when recombination rates are low, background alleles may hitchhike with adaptive alleles, resulting in the fixation of background alleles between habitats. Given sufficient recombination, background alleles will eventually become randomly distributed across niches (‘mixed’), making them unlikely to be confused for adaptive loci. We defined a population as ‘mixed’ when background alleles were randomly distributed across niches (*e.g.* background allele 1 at frequency 0.5 in both niches). We also defined *t(mix)* as the time, in generations, when mixing is achieved in a simulation initiated as described in section 2 above, with the derived species at an initial frequency of 1%, and background alleles in perfect association with niches (allele 0 in niche 0 only; allele 1 in niche 1 only).

As expected, *t(mix)* was highly dependent on the recombination rate. Under additive fitness and *s = *0.01, as the recombination rate increased, less time was required to achieve mixing: the mean *t(mix)* across replicate simulations was always over 100,000 generations with *r* = 10^−5^, 7,999 generations (s.d. = 2420) with *r* = 10^−4^, and only 62 generations (s.d. = 2.2) with *r* = 10^−2^. This suggests that at high recombination rates, adaptive loci should be easily discernible from neutral loci very soon after the initiation of speciation, but at low to moderate recombination rates neutral loci may hitchhike for thousands of generations, making them easy to confuse with adaptive loci using many standard population genetic tests for positive selection [43].

## Discussion

Our simulations identified a major tradeoff between early- and late-stage recombination, predicting that the initiation of sympatric speciation is much more likely when the number of loci required to adapt to a new niche is small. The same qualitative result is obtained using different selection models and selection coefficients. As the number of loci increases, the tradeoff becomes more restrictive: high recombination rates are required to generate multi-locus optimal genotypes at early stages, but such high recombination rates eventually homogenize gene pools and prevent the maintenance of ecologically adapted species at late stages. This result, which is qualitatively similar to what occurs in sexual populations, is obtained in a model of asexually-reproducing bacteria with no assortative mating.

Have we described a realistic model of bacterial sympatric speciation? It is certainly not a model of biological species because biological speciation is impossible without barriers to recombination, which are not included in our model. It is more a model of early niche invasion and evolution of optimal genotypes. We argue that this is an important early step toward speciation, and one that is increasingly observed in diverging natural microbial sympatric populations, which mix by recombination at all but a few adaptive loci [1], [41]. Because the fitness landscapes of these diverging populations are unknown, we have used two very different fitness models, one additive and one ‘step’, which can be considered an extreme version of epistasis. Other fitness models are also plausible, so long as they include some degree of disruptive selection [7], but a thorough investigation of other models is beyond the scope of this study. Despite being a deliberate abstraction of real fitness landscapes, our model describes one simple (but not exclusive) mechanism that generates data consistent with the observation that relatively few adaptive loci tend to drive early stages of sympatric speciation, in microbes just as in many sexual eukaryotes [27], [33], [45], [46].

Another limitation of our model is that extinction occurs when any of the adaptive alleles disappears. In real bacterial populations, alleles may be replenished by recombination from other populations, giving speciation another chance to occur. Therefore, our model probably overestimates the likelihood of extinction preventing speciation. Due to this and other limitations and assumptions, we do not claim that there is a single ‘magic number’ of adaptive loci that provides the easiest path to sympatric ecological speciation. The exact number will always depend on the recombination rate, selection strength, population size and niche complexity, but smaller numbers will always tend to facilitate speciation.

Given the tradeoff predicted by our simple model, how do pairs of well-adapted sympatric bacterial species emerge? Perhaps they do not, or only rarely. Ecological species may be transient, and rapidly merge back in to a homogenous parent population. In cases where a new species does evolve, the ‘effective’ number of adaptive loci could be kept low by combining suites of genes into operons, allowing complex phenotypes to be acquired in a single recombination event. Our model could thus provide an example of how linking coadapted genes together into operons, in addition to providing a selective advantage for the genes themselves – as in the selfish operon hypothesis [47] – could also provide an advantage in facilitating ecological speciation. Despite such ‘strategies’ to keep *L* low, it is possible that many opportunities for speciation are missed because new niches are too complex to be exploited by genotypes with just a few adaptive alleles. However, there is mounting evidence from natural microbial communities that surprisingly few adaptive genes or alleles may be sufficient for adaptation to fairly complex niches, including different hosts or nutrient utilization strategies [1], [41], [48], [49]. In other cases, large ‘continents’ of divergence have been observed, containing many genes [40]. Such continents may not fit the model described here, or may come about at later time points in the speciation process, perhaps once gene flow has become more restricted.

What if many (>>10) adaptive loci are required to exploit a new niche? Perhaps speciation could still be achieved if recombination rates varied over time, such that the stress experienced in a new habitat would trigger periods of hyper-recombination, promoting the rapid generation of genotypes with the optimal combination of adaptive alleles. In order for new species to be maintained on the long term (rather than being eroded by recombination with the ancestral species), recombination would have to revert to a low rate. This could occur if barriers to gene exchange between niches, or forms of assortative mating, emerged over time.

It is known that sex (recombination) can slow the rate of adaptation within a population when the fitness landscape is such that alleles must be acquired in a particular order to be adaptive [23]. Here we have shown another intuitive, yet to our knowledge unrecognized, disadvantage of sex that arises even in simple, additive or step fitness landscapes without assortative mating. Recombination, although advantageous in the early stages of speciation, erodes ecological specialization later on, resulting in less fit populations. As a result, recombining microbial populations, like sexual eukaryotic populations, are predicted to form new species using only a few loci. Although this limitation is common to microbes and sexual eukaryotes, it comes about for different reasons. In simulated populations of sexual eukaryotes, recombination is uniformly high (occurring before every mating), and some form of assortative mating is usually required for speciation (*e.g.* [16]). In the earlier models of Kondrashov [3], [4], [5], [8] and Felsenstein [16], it was shown how the reduced efficacy of selection with increasing number of loci (involved in adaptation, assortative mating, or both) is important in preventing the initiation of sympatric speciation, and also how recombination later erodes ecological specialization. Here, we have presented an updated model, specifically for microbial populations with clonal reproduction, variable recombination rates, and without assortative mating. In microbes, although the efficacy of selection is still important, the recombination rate is the key factor: for ecological traits controlled by many adaptive loci, high recombination rates are necessary to generate a new species, but this also produces many intermediate genotypes and reduces the completeness of speciation at later stages. A simple, although not exclusive, solution to this tradeoff is to require adaptation at just a few loci to initiate and maintain ecological speciation. The generality of this solution will be put to the test as more data become available from population genomic studies of ecologically diverse, recombining wild microbial populations.
